# Elder abuse and neglect in the midst of COVID-19

**DOI:** 10.7189/jogh.11.03122

**Published:** 2021-11-20

**Authors:** Raudah Mohd Yunus, Nik Nairan Abdullah, Muhammad Abbas M Firdaus

**Affiliations:** 1 Department of Public Health Medicine, Faculty of Medicine, Sungai Buloh Campus, Universiti Teknologi MARA, (UiTM), Malaysia; 2 Faculty of Medicine, Sungai Buloh Campus, Universiti Teknologi MARA (UiTM), Malaysia

Historically, large-scale crises have been associated with more occurrences of family and interpersonal violence. Using empirical data, Catani et al demonstrated in 2008 a relationship between war violence in Sri Lanka (during the civil war) and infliction of violence on children by the family [1]. On the other hand, Rezaeian conducted a systematic literature review in 2013 and argued that natural disasters are associated with increased interpersonal violence, in which spouses, children or older adults can be victims [2]. Older adults are also said to be more vulnerable particularly during humanitarian crises given their unique characteristics and special needs that often go unrecognized [3]. All these indicate that national or global crises – be they political or economic or social or health – can create situations ripe for violence and maltreatment within families as a result of negative coping mechanisms (eg, outward expression of anger and stress) among individuals [2].

Other than COVID-19-related morbidity and mortality, the vast majority – including older adults – have suffered more from the measures taken to contain the pandemic. These include national lockdowns, movement restrictions, closures of schools and non-essential services, and economic shutdowns. The actual extent of the impact of these countermeasures is difficult to determine, but emerging evidence shows that it is multi-faceted and recovery will require a long time. No age group has been spared from the effects of COVID-19 containment measures; millions of adults in the labour force have been laid off, women have endured increasing domestic violence (DV), children have suffered from educational disruption and limited outdoor exposure, and older adults are put at higher risks of social isolation in the community and institutional settings.

## COVID-19 AND ELDER ABUSE

Worldwide, reports have shown that domestic violence has been on the rise since the pandemic struck [4]. This could be attributed to various reasons such as stay-at-home orders that force victims to stay in close proximity to abusers, social distancing measures that render vulnerable women and children socially isolated thus having less chance to seek help, cessation of non-essential services that restricts DV victims’ access to aid, and the negative economic impact that causes anxiety and household tension. When applied to older adults, all these factors are likely to amplify as old age is often compounded by chronic diseases, physical limitation, dependency on caregivers and institutionalization.

**Figure:**
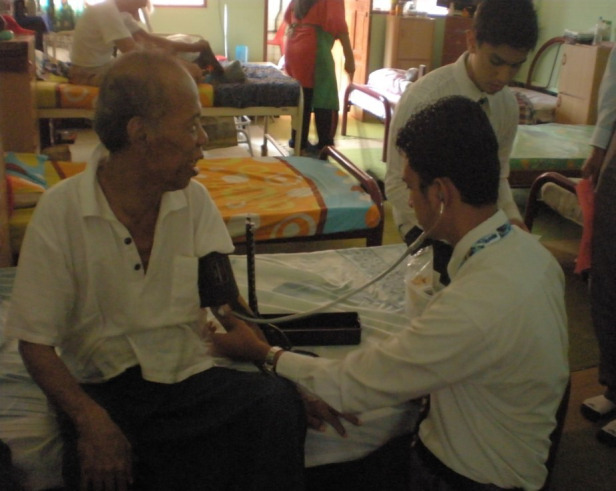
Photo: Older adults living in nursing homes receive regular visitors prior to the pandemic. However, they have been kept in relative isolation once COVID-19 struck, to avoid risks of transmission. From Nik Nairan’s collection, used with permission.

A recent study on elder abuse and neglect (EAN) in Hunan province in China reported a prevalence of 15.4%, higher than the pre-pandemic Figure [5]. Another study in the United States found EAN prevalence at 21.3%, a huge leap from figures prior to COVID-19 [6]. Likewise, in a survey among older adults across 17 states and 4 Union Territories in India, 71% believed abuse was on the rise, while 56.1% and 63.7% respondents reported experiencing abuse and neglect, respectively [7]. Robust data from other low- and middle-income countries (LMICs) remains scarce, but circumstantial evidence hints at an overall upward trend of family violence [8,9]. For instance, while little is known about EAN patterns in Malaysia during the pandemic, media and government sources documented a huge increase in the number of hotline calls and requests for assistance by victims of family violence [10]. Even though in Malaysia, the notion of family violence typically refers to victimization of women and children, this reflects increasing tension and conflicts within households or worsening family dynamics as a result of the stresses related to COVID-19 prolonged countermeasures – all of which would affect older parents directly or indirectly.

## DYNAMICS OF ELDER ABUSE IN TIMES OF CRISIS

The dynamics of EAN in times of COVID-19 are diverse and complex. This health crisis can trigger EAN by affecting older adults, caregivers and the caregiving context [11]. For older adults, social distancing measures and movement restrictions predispose them to social isolation and loneliness more than other age groups. This is because many older adults reside in rural areas and derive physical and social support from frequent visits by children and relatives. When social visits and gatherings are banned, they are more likely to feel the negative impact of this measure. Loss of support among older adults who have limited physical function or specific medical needs can easily compromise their safety and health, and make them victims of unintentional neglect. Nursing home residents are at even greater risks of isolation and loneliness as visitors and visiting hours are restricted in order to avoid disease transmission. In addition, communal activities that are regularly run by nursing home operators to enable older adults to participate in public life and derive pleasure from social interactions have been largely stopped for fear of exposure to COVID-19 [12].

For victims of EAN who co-reside with perpetrators, being forced to stay at home poses an additional risk. The ability to go out and interact with peers and participate in outdoor activities are important to build social capital, which is a buffer against social isolation and maltreatment. Deprivation of social interaction and physical contact with friends makes it easier for abusers to control, manipulate and mistreat older adults as there would be less interference and informal surveillance, thus lowering chances for detection. Similarly, older adults would find it more difficult to reach out to their other family members or acquaintances to seek help as their movements are curtailed. Despite the rapid growth and uptake of digital technology and virtual platforms of communication in the midst of this global pandemic, such means are not necessarily available or convenient to older adults. Even when availability is not an issue, older adults are generally less aware and less capable of making use of them compared to the younger age groups [13].

Among the impacts of the COVID-19 pandemic is disruption of medical and health-related services as resources are channeled to COVID-19 control and treatment. Older adults may find their scheduled appointments cancelled or repeatedly delayed, and their access to medications for chronic illnesses interrupted [14]. Similarly, other health-related and long-term care services such as physiotherapy, rehabilitation, and assistance with activities of daily living (ADL) might be temporarily halted. This could be due to a change in policy by service providers to reduce the risks of disease transmission, or inadequate manpower when many health care and social workers have contracted COVID-19 or are put under quarantine. In addition, these health care workers may have been deployed to the care of COVID-19 patients. As a result, older adults may find their health and living conditions rapidly deteriorating – all of which comprise important risk factors for EAN.

The economic downturn that has affected thousands of businesses and millions of families, along with the lack of an adequate safety net, can provide a fertile ground for financial abuse and exploitation [15]. From one perspective, older adults may see their retirement or investment schemes dwindle as the local and global economies slow down [16]. On the other hand, adult children who have been plunged into financial distress and uncertainty may resort to their parents for assistance [5]. While the general assumption is that help within families is based on mutual understanding and filial love, it is difficult to rule out possibilities of financial coercion and manipulation. Older adults who receive regular pension or monetary aid can be targeted by family members, while those with properties can be forced or deceived into selling them or transferring ownership of these properties to others. Another type of elder financial abuse that has been less studied – exploitation by strangers in the forms of online fraud and scams – has also been reported to increase in the aftermath of COVID-19 [17].

Informal caregivers of older adults are generally spouses or adult children or extended family members. Many of them juggle between providing care for older parents and maintaining a job, on top of caring for their own young children. Closure of schools and economic instability predispose caregivers to higher levels of stress, as their workload now extends to caring for school-aged children and having to cope with job insecurity or loss of income. This can easily lead to physical and emotional burnout, an important factor for EAN [18]. Moreover, caregivers with a history of substance abuse may resort to alcohol or drugs as a coping mechanism – another condition that puts care recipients at risk of abuse. Access to services for individuals struggling with alcoholism and drug addiction in turn, may be restricted in times of COVID-19, as health priorities shift.

Beyond victims, perpetrators and the caregiving context, the wider structural determinants of EAN need to be thoroughly and equally scrutinized, in order for us to understand how they interact with risks of abuse and contribute to, or protect against, EAN. One prominent example is ageism. From the beginning of the pandemic, the older population has been put under the spotlight for numerous reasons, most of which are negatively portrayed. Despite enduring higher risks of morbidity and mortality, interventions by public health authorities have not been sensitive enough to older adults’ needs and well-being [19]. Ample evidence shows that during COVID-19, ageist language and attitudes have become more pervasive, infiltrating the conventional and social media, public discourse and even the health care sector [20]. Public discussions and media reports seem to have implicitly reinforced the notion of older adults being a burden to society and that their lives are less valuable compared to the young. Even though the link between ageism and EAN is still being debated, emerging evidence suggests a relationship between the two [21]. We constructed the following diagram (**Figure 1**) to illustrate the dynamics of the impact of COVID-19 countermeasures on risks of EAN.

There are other systemic factors that enable EAN in the midst of this pandemic, such as the lack of national response to specifically cater for older adults’ needs, interruption of social and welfare services which typically respond to EAN, and cessation of social platforms or ‘safe spaces’ that are often used by older adults and act as a buffer against EAN. Likewise, little is known about disruptions of long-term care services and how these might have contributed to cases of neglect and abandonment. ‘Protective’ measures taken by, or imposed on, nursing homes have been inadequately studied and understood. Whether they are effective in reducing contagion at the expense of greater risks of abuse is a question to which we have no answer yet.

## CONCLUSION

In order to understand the full impact of a health crisis like COVID-19 (and its countermeasures) on EAN, a holistic approach is needed. Factors that affect all the eco-systemic levels of EAN risks need to be taken into account, including those at the victim level, perpetrator level, victim-perpetrator relationship level, family level and the wider community or socio-cultural level. Existing studies have focused more on risks related to victims, but this pandemic has laid bare the multiple layers of risks and factors causing EAN. This should prompt researchers, social workers and policy-makers to adopt a different approach in understanding EAN and apply a more comprehensive framework of thinking while designing intervention and preventive measures.

**Table:** **Figure 1.** Framework on the relationship between COVID-19 counter-measures and elder abuse and neglect.
